# ‘When a patient chooses to die at home, that's what they want… comfort, home’: Brilliance in community‐based palliative care nursing

**DOI:** 10.1111/hex.13780

**Published:** 2023-06-09

**Authors:** Ann Dadich, Michael Hodgins, Kerrie Womsley, Aileen Collier

**Affiliations:** ^1^ School of Business Western Sydney University Parramatta New South Wales Australia; ^2^ School of Clinical Medicine University of New South Wales Sydney New South Wales Australia; ^3^ Palliative Care Service Illawarra Shoalhaven Local Health District Wollongong New South Wales Australia; ^4^ Faculty of Medical and Health Sciences University of Auckland Auckland New Zealand

**Keywords:** brilliant care, community health, knowledge translation, palliative care, positive organisational scholarship, teleological analysis, video‐reflexive ethnography

## Abstract

**Introduction:**

To redress the scholarly preoccupation with gaps, issues, and problems in palliative care, this article extends previous findings on what constitutes brilliant palliative care to ask what brilliant nursing practices are supported and promoted.

**Methods:**

This study involved the methodology of POSH‐VRE, which combines positive organisational scholarship in healthcare (POSH) with video‐reflexive ethnography (VRE). From August 2015 to May 2017, inclusive, nurses affiliated with a community health service who delivered palliative care, contributed to this study as co‐researchers (*n* = 4) or participants (*n* = 20). Patients who received palliative care (*n* = 30) and carers (*n* = 16) contributed as secondary participants, as they were part of observed instances of palliative care. With a particular focus on the practices and experiences that exceeded expectations and brought joy and delight, the study involved capturing video‐recordings of community‐based palliative care in situ; reflexively analysing the recordings with the nurses; as well as ethnography to witness, experience, and understand practices and experiences. Data were analysed, teleologically, to clarify what brilliant practices were supported and promoted.

**Results:**

Brilliant community‐based palliative care nursing largely involved maintaining normality in patients’ and carers’ lives. The nurses demonstrated this by masking the clinical aspects of their role, normalising these aspects, and appreciating alternative ‘normals’.

**Conclusion:**

Redressing the scholarly preoccupation with gaps, issues, and problems in palliative care, this article demonstrates how what is ordinary is extraordinary. Specifically, given the intrusiveness and abnormalising effects of technical clinical interventions, brilliant community‐based palliative care can be realised when nurses enact practices that serve to promote a patient or carer to normality.

**Patient or Public Contribution:**

Patients and carers contributed to this study as participants, while nurses contributed to this study as co‐researchers in the conduct of the study, the analysis and interpretation of the data, and the preparation of the article.

## 1 INTRODUCTION

Palliative care is understood to be ‘an approach’ to improve the quality of life among patients with life‐limiting illness and their carers.^1^ It involves addressing physiological, psychosocial, and spiritual needs.

Literature suggests that the demand for palliative care is rising, given ageing populations^2^ and the prevalence of chronic health issues.^3^ International projections^4^, ^5^ suggest that palliative care will chiefly be needed by people aged 85 years and older–furthermore, the proportion of people dying from multimorbidities will increase by 60% by 2040. Yet there are barriers to accessing (quality) palliative care, particularly at home.^6^ These barriers are associated with personal,^7^ social,^8^ and economic implications.^9^ Palliative care, therefore, needs to be recognised as an approach that involves different sectors^10^ to address issues about access and, relatedly, the negative views about palliative care, which are evident in research^11^, ^12^, ^13^, ^14^, ^15^, ^16^ and the media.^17^

This literature depicts a somewhat bleak portrayal of palliative care. While it is important to identify gaps, issues, and problems, a preoccupation with what is wrong with healthcare can be problematic. Such negative discourse can silence patients’ and carers’ positive experiences with health issues and/or health services—and there are many instances of these^18^; it can also diminish help‐seeking behaviours and access to timely care.^19^ For nurses and service managers, this preoccupation risks unfairly stereotyping these practitioners as part of a systemic problem^20^—for instance, in an international study, those who delivered and managed community‐based palliative care described variations in care, partly due to poor funding.^21^ And for policymakers, the negative discourse might continue to direct their attention and public funds to ineffective and/or inefficient healthcare practices. This is because, rather than problematise beliefs and assumptions, the identification of problems, gaps, and issues is largely based on prevailing beliefs and assumptions, leaving little opportunity for innovation.^22^

To redress the scholarly preoccupation with gaps, issues, and problems in palliative care, and extend previous findings on what constitutes brilliant palliative care,^23^ the aim of this study is to clarify what brilliant nursing practices supported and promoted. Brilliant care is not tied to specific health outcomes. It is a relational experience that exceeds expectations, bringing joy and delight to those who experience or witness it.^24^ Brilliant care can be unconventional, serendipitous, and does not necessarily represent business as usual within a service or a sector. While brilliant care is largely in the eye of the beholder, it is interpersonal, uplifting, inspiring, and/or energising.^25^

Conceptually, brilliant care draws on two complementary areas of research—namely, Fredrickson's^26^ broaden‐and‐build theory; and an ethic of care perspective.^27^ The former suggests that ‘everyday positive emotions, as fleeting as they may be, can initiate a cascade of psychological processes that carry enduring impact on people's subsequent emotional well‐being’.^28^ The latter ‘recast[s] the conversation about self and morality as a conversation about… relationships’.^29^ Undergirded by ‘a psychological logic of relationships', rather than a ‘formal logic of fairness’,^27^ it recognises the importance of ‘trust and responsibility, protection of individuality, the context in which the relationship takes place, and the quality of the relationship’.^30^ Furthermore, it recognises listening as a way to fortify trust, strengthen relationships, and foster empathy.

Research on brilliant palliative care suggests that it involves: ‘anticipatory aptitude and action; a weave of commitment; flexible adaptability; and/or team capacity‐building’.^23^ However, what remains unknown is what brilliant nursing practices helped to support and promote—thus, this study addresses this focus.

## 2 METHODS

This study involved positive organisational scholarship in healthcare (POSH) and video‐reflexive ethnography (VRE).^31^ POSH is ‘the study of that which is positive, flourishing, and life‐giving in [healthcare] organisations’.^32^ Challenging the tendency to concentrate on all that is negative, it seeks to study triumphs and achievements because of their inherent allure.^33^ Given that POSH is underpinned by critical theory, it does not ignore negative organisational aspects^34^; but rather, it represents ‘an alteration in focus’^32^—a deliberate attempt to redress the preoccupation with the nonpositive.

VRE involves inviting participants to the following: feature in and/or gather visual data (V); interpret the data by examining and shaping situations as they unfold^35^ (R); and use different research methods to suspend and understand practices and experiences in situ (E). VRE thus represents a powerful channel to inspire change^36^—this is because video‐recordings can attune people to personal and interpersonal dimensions they might not otherwise have considered^37^—consider times when you might have viewed yourself in a home video, only to realise happenings that you did not recall or even witness at the time. Combining the approaches, POSH‐VRE was used to clarify what brilliant nursing practices supported and promoted.

### 2.1 Participants

The study involved co‐researchers who delivered palliative care as a specialist palliative care nurse or a generalist community health nurse (*n* = 4). The academic researchers trained and supported these individuals to build their research capacity. Participants included the following: nurses who delivered palliative care (*n* = 20); patients who received palliative care (*n* = 30); and unpaid carers (including family members) of persons receiving palliative care (*n* = 16; see Table 1).

**Table 1:** Demographic information.

	Academics (*n* = 3)	Co‐Researchers (*n* = 4)	Participants (*n* = 20)
Gender			
Female	2	4	17
Male	1	0	3
Position			
Senior academic	2	0	0
Junior academic	1	0	0
Clinical nurse consultant	0	1	0
Clinical nurse specialist	0	1	2
Registered nurse	0	2	18

### 2.2 Setting

Data collection occurred at a community health centre that offered different health services to the community, including palliative care (see Table 2). The centre was identified via consultation with senior nurse managers who were invited to consider the palliative care services within their local health district that had developed a reputation for exceptional care. Following this, a meeting was held with the nurses and their managers at the nominated centre to invite their involvement in the study.

**Table 2:** Setting.

Located in an area of New South Wales with a proportionally high population of culturally and linguistically diverse residents,^38^ data collection occurred at a community health centre that offered different health services to the community, 7 days a week. Funded by the state government, these included child and family services, acute and post‐acute treatment, as well as palliative care. In addition to delivering palliative care directly to patients and carers, a small team of specialist palliative care nurses, led by a clinical nurse consultant, provided expertise, mentorship, and support to a larger team of generalist community health nurses. Palliative care was typically delivered in patient homes. Each week, the specialist palliative care nurses, a visiting palliative care medic (who also visited patients in their homes, as required), and the generalist community health nurses met to review patients under their care. The purpose of the reviews was to assess patients’ situation and revise their treatment and care plans as required. In addition to a discussion of a patient's clinical situation, the reviews would typically include a discussion of their psychological and social situation, and the potential value of additional services, such as social work, occupational therapy, and rental services from which hospital beds, oxygen concentrators, and other equipment could be hired.

### 2.3 Data collection

Over approximately 21 months (August 2015 to May 2017, inclusive), and following participants’ informed consent, the researchers and co‐researchers used POSH‐VRE by capturing video‐recordings of community‐based palliative care, in situ; reflexively analysing the recordings with the nurses (both the co‐researchers and participants) to identify the practices and experiences that exceeded expectation and brought joy and delight as well as clarify what enabled and sustained them; and ethnography (without using a video‐recorder) to understand palliative care. Video‐recording practices involved capturing typical and atypical practices^25^—the former included routinised behaviours or acts that reflected protocols or organisational norms, while the latter included observances that were not necessarily dictated or influenced by protocols or organisational norms. These included weekly case reviews; team discussions; the delivery of palliative care within patient homes; conversations between nurses, with patients, and/or with carers; and the documentation of clinical notes. Although the section on data analysis clarifies how brilliant palliative care was understood, there was limited understanding of when and how brilliance might manifest, because, as noted, it is largely in the eye of the beholder—as such, close to 60 h of digital video files were recorded (54.93 h), some of which might not have captured brilliant palliative care. Reflecting situated ethics,^39^ and as described elsewhere,^25^ decisions about what was recorded, when, and how were determined with the nurses, patients, and their carers. The video‐recording only occurred when those who were (or might be) in front of the camera lens approved it.

### 2.4 Ethical considerations

Following clearance from the relevant ethics committees (reference number: HREC/15/LPOOL/73), clinicians affiliated with a community health centre in New South Wales, Australia, who delivered palliative care, were invited to contribute to this study as co‐researchers or participants.

The conduct of this study was informed by both procedural ethics, as per national guidelines,^40^ and situated ethics.^39^ Thus, although informed, written consent was obtained from all participants, participants were regularly invited to consider the following: whether they wished to continue their involvement; how; and the associated implications. To respect participant anonymity, data use is devoid of personal identifiers.

### 2.5 Data analysis

Throughout this study, the researchers and co‐researchers developed a shared understanding of brilliance.^25^ This involved discussing and clarifying the overarching purpose of palliative care, and what is and is not brilliant palliative care nursing (see Table 3), whereby brilliance is a relational experience that exceeds expectation, bringing joy and delight to those who experience or witness it.^23^, ^25^ Although not digitally recorded, this dialogue continued throughout the study, informing the analysis and the understanding of brilliant palliative care nursing. This is not to suggest that recognising and defining brilliant palliative care nursing was easy—the researchers and co‐researchers regularly engaged in healthy debate, respectfully critiquing and contradicting each other's understandings. They considered the dimensions that gave rise to and sustained brilliance—the dimensions that enabled individuals and collectives to have extraordinary experiences. For comparative value, this also required consideration of experiences that were less than brilliant—those that were mediocre, uninspiring, disappointing, and frustrating, among others.^43^

**Table 3:** Data analysis.

1. The researchers and co‐researchers:
1.1. Discussed and clarified the purpose of palliative care, as
1.1.1. Articulated by government departments^41^
1.1.2. Constructed by evidence‐based practices^42^
1.1.3. Embodied by the nurses, daily
1.2. Clarified what is and is not brilliant palliative care nursing, whereby brilliance is a relational experience that exceeds expectation, bringing joy and delight to those who experience or witness it^23^, ^25^
1.3. Reviewed video‐recordings
1.4. Identified instances of brilliant palliative care nursing
1.5. Edited the footage into video clips that did not sever the exemplar from its context (average length: 3.50 min)
2. The co‐researchers and nurses participated in 4 reflexive sessions to analyse the short video‐recordings—this involved gathering to discuss:
2.1. What they observed
2.2. How they felt while viewing the footage
2.3. Whether and why the exemplar epitomised brilliant palliative care
2.4. Factors that influenced this moment
3. The researchers and co‐researchers:
3.1. Analysed video‐recordings of the reflexive sessions
3.2. Documented when brilliance was recognised and discussed—specifically:
3.2.1. How brilliance manifested
3.2.2. Why
3.2.3. The dimensions that gave rise to and sustained brilliant community‐based palliative care
3.2.4. What brilliant practices supported and promoted

**Figure 1:**
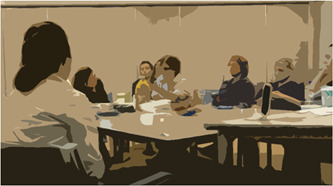
A reflexive session.

Informed by this understanding of brilliant palliative care nursing, co‐researchers and nurses participated in four reflexive sessions to analyse select exemplars of brilliant palliative care (see Figure 1). In preparation for these sessions, the researchers and co‐researchers selected footage that epitomised brilliance. Given the absence of a checklist, they reviewed video‐recordings, identified instances they deemed to depict brilliant palliative care nursing, and edited the footage into video‐clips that did not sever the exemplar from its context.

Framed by POSH, the reflexive sessions—which were video‐recorded—encouraged the researchers, co‐researchers, and nurses to critically reflect on their role as a researcher and/or a nurse and isolate teachable moments that epitomised brilliance (see Table 3). The video‐recorded reflexive sessions were then analysed to understand nurse perceptions of brilliant community‐based palliative care. This involved reviewing the footage and documenting instances when the co‐researchers and nurses recognised and discussed brilliance.

For this article, a teleological analysis of brilliance was pursued by exploring what brilliant practices supported and promoted. Teleology is the study of phenomena in terms of the purpose they serve, rather than of the cause by which they arise.^44^ It serves to direct inquiry to ‘an actor's purposeful, planned action… [or] conscious goal‐orientation toward a defined aim’.^45^ A teleological approach required us to question the theoretical endpoint of the data about brilliant community‐based palliative care nursing—to ask what did this brilliance accomplish?

### 2.6 Rigour

To determine the credibility of the findings, footage of the reflexive sessions was supplemented with additional ethnographic data—namely, footage of brilliant palliative care in situ, fieldwork notes, and opportunistic interviewing with staff members at the community health centre. The use of these data enriched the perspectives of brilliance. These two phases of analysis—namely, the reflexive sessions and the analysis of the reflexive sessions with reference to supplementary data—optimised the rigour of this study. They enabled the following: triangulation and thick description through the comparison of different kinds of data related to a single finding or theme; prolonged engagement with the context of community‐based palliative care and the brilliance, therein; peer debriefing and member checking within the research team to ensure the validity of findings and avert the potential negative impacts of fieldwork; and reflexivity through, for instance, the use of reflexive fieldnotes, which situated the researchers’ perspectives and worldviews in the context of findings.^46^

## 3 RESULTS

### 3.1 Masking clinical practices

Normality might be considered to contrast with brilliance and perhaps clinical practice, generally. Clinical practices do not typically focus on maintaining a status quo, but are interventional, treating patients out of sickness. Conversely, brilliant palliative care often appeared to relegate clinical practices and foreground caring relationships. Even when clinical practices were present, there was a brilliance to how the nurses hid these in mundane behaviours. Consider a nurse who made a Movicol ‘milkshake’ to give to a patient to ease his constipation. As other nurses analysed a video‐recording of the event during a reflexive session, they commented on how comfortably the nurse fitted within the home environment:

Watching that video, if you took the sound off or if you weren't from a medical or nursing background, you wouldn't know that she was making something that was a treatment; it would look like you were making a cup of tea. And I think that's the difference… when a patient chooses to die at home, that's what they want. Take out the clinical… [It's] comfort, home.

The removal of the ‘clinical‐ness’ from this action was important to the nurses—they identified how clinical actions can contrast the comfort and safety of a patient's home environment. They also commented on the trust and continuity the image represented:

There's a trust factor there, you can see it… She looked comfortable… It looked like she'd been there like months… Even you preparing that drink, there was no question of, ‘Are you sure you know what you're doing? Do you know what you're putting in there? Oh, I'm a little bit sceptical’… Whatever it is you were going to give him, he was happy to take it.

Instead of concentrating on the clinical importance of the treatment, the nurses were interested in how well the treatment fit within a normal context. In the aforementioned excerpt, the nurses also highlighted the confidence with which the pictured nurse carried herself within the patient's home.

Another example of hiding clinical practices under a veil of normality was the verbal assessments of patient symptoms. As part of home visits, nurses assessed patients to determine how they managed their illnesses and symptoms. These assessments covered anything from a patient's pain to their social circumstances. The nurses often spoke about their assessments starting as soon as they entered a patient's home:

As soon as you walk into the patient's house, there goes your assessment. Your assessment already started—by the way you look at the patient, how they walk, how they stand from the chair, and how they walk from the chair and from the lounge room to the kitchen.

The nurses rarely explicitly informed patients about these assessments during a home visit. This contrasted with medical members of the team who were usually more explicit about their patient assessments, as they informed patients they were ‘going to have a look at how [patients were] getting around’, for instance. Conversely, the nurses rarely described their assessments. During one reflexive session, as a nurse's assessment of a patient was pictured, the nurses reflected on how it seamlessly fit within a conversational tone:

it's… that interaction… you're sort of asking questions as you went along… it flowed really well… even though you're asking questions… You're doing it in a way that nobody really realises or can see exactly why you're asking. It's just like a normal conversation between two people, but you're getting all the information that you need.

The nurses spoke of disliking apparatuses that inhibited this conversational tone as part of the assessment—specifically, the computer tablet they were required to use to record notes. Many refused to use the tablet during home visits, claiming it impeded interactions:

I have a really big thing for sitting down with clients and actually talking to them. I know there's a big thing about the computers, I think they're great. But… it's like, you go and see the GP [general practitioner] and they say, ‘So, what's wrong with you? You look okay’ [fingers tap on table]—‘Here's your script’. There's no conversation; there's no rapport—and so, having the computer there, I don't think is always for the benefit of the client.

The nurses would go to great lengths to avoid impeding patient interactions. One participant spoke of waiting until late in the evening to document case notes to ensure their practices were as ‘non‐inhibitory to the interaction as possible’. This instance represented a common theme among the nurses’ discussion of interactions during home visits—that of maintaining flow and attention. An obvious reading of this theme is the importance of capturing vital patient‐provided information and treating patients respectfully. But, in the context of the broader analysis of normality, attention to these interactions also represented the importance—in the nurses’ eyes—of normal interactions with patients.

According to the nurses, another aspect of brilliance was the ability to provide a calm, confident front for patients and carers as this normalised relationships. It required the nurses to subjugate feelings related to personal and organisational challenges. As demonstrated during home visits, this was represented by a friendly and polite interaction with a patient—or, as some put it, becoming a ‘person’ rather than a ‘nurse’:

It's… like taking off your skin but… instead of them becoming the clinician, it's becoming that person… it was that empathy, that understanding.

Despite their appearance during home visits, the nurses often preceded or followed these interactions with debriefs in the fleet vehicle with an academic researcher where they released their frustrations. This might have alleviated some tension, before their next patient visit.

### 3.2 Normalising interventions

The nurses were often unable to completely mask the clinical aspects of their role to patients. Technical clinical interventions were a necessity to community‐based palliative care—for example, the presence of syringe drivers, which gradually delivered medications to patients over 24 h. Interventions like these could not be masked; instead, the nurses focused on normalising these kinds of clinical intrusions in a patient's home. One example involved the Bristol stool scale. This scale helps to gauge the appearance and consistency of bowel movements, enabling nurses to identify whether patients experienced diarrhoea or constipation. In this excerpt of a case review, a nurse commented on how the scale became a consistent part of patient interactions:

I… gave her a Bristol scale and… she goes, ‘Yep, it's like that!’… it's the only person I've seen that actually uses it and is quite adamant about what it is… ‘It was like that today… but I think, it should be like that, shouldn't it?’ and I go, ‘Yeah’… she was very engaged in what we were talking to her about.

The nurses were typically overjoyed when patients took it upon themselves to incorporate clinical interventions into their normal routines.

### 3.3 Continuing care

The nurses recognised the importance of continuity of care. This continuity was represented by multiple nurses, able to work with a single patient and continue the established relationship with the service:

If I'm the first one to go in, then hopefully when somebody else goes in, that rapport will just continue—so, it's about making sure the team's okay with what's been said.

Establishing rapport was represented by spending time, having easy and positive interactions with patients, and developing trust by balancing optimism and honesty. These elements of palliative care are well‐represented in the literature.^47^ However, it is important to also consider the purposes of rapport building. The nurses suggested that rapport‐building practices represented an ‘investment’ in the continuity of care. In the context of this analysis, implicit in this continuity of care was the normalising of a clinical presence in the home, no matter who it was:

the wife, said to [her husband]… ‘She's with [the nurse]’—‘Oh! So that's okay’… You going in and not making him feel uncomfortable means everybody else behind you is okay.

Normalising ‘the nurse’ as a presence in a home and ensuring the presence was as ‘comfortable’ as possible, meant the nurses could handover patients with ease if they were on leave. When the nurses spoke of teamwork in the context of brilliance, this was often in relation to this ability to enable interchanging patients.

Part of the brilliance the nurses identified in their own practice was understanding how to maintain a balance between their clinical presence in the home and allowing patients to live unimpeded, normal lives. One way this was represented was by keeping patients ‘on the books’. Given the need to manage limited resources efficiently, the nurses were often required to justify why they visited patients who required less technical clinical care. The nurses spoke of the need to maintain a connection with patients, knowing a ‘crash’ or drastic increase in clinical need could come at any time:

When he was good, he's good, and then when he crashed, he'd been in pain and then you just couldn't get on top of it. So, you could have these periods of like, ‘I haven't seen you for six weeks’ and she's saying, ‘We're fine. Go and see somebody else’. ‘Oh, I have to see you or the boss is going to discharge you’. ‘You tell that boss!’… But sometimes they are travelling well, and they don't want you in their face every four weeks, but you need to keep them on the books.

At times, the nurses noted the need to limit their contact with patients to avoid unnecessarily infringing on their lives. This was considered positive if the nurses could recognise when and how to increase their contact with a patient.

### 3.4 Respecting patients' and carers' personal agency

Central to maintaining normality for patients and carers was the nurse's ability to respect patients' and carers' personal agency. One way this was represented was the nurse's praise of a patient's ability to function in the often‐challenging environments of their home. Patients' home environments were diverse and sometimes presented novel challenges for the nurses. They sometimes worked in unsanitary conditions; dealt with patients and carers who were aggressive; travelled to remote, hard‐to‐reach destinations; and negotiated patients' and carers' unsafe or illegal behaviours. Accepting these environments and avoiding judgement was cited as an important aspect of community‐based care:

It can come down to… ‘Where am I going to put the stuff?' as you walk in the house… and I only have this much room and I need this much or… the bed's here or there's no light and I can't see anything. All that stuff… you don't think about until you actually come out in the community.

Beyond just functioning in these environments, the nurses noted the ability to empathise with patients' and carers' lived experiences—or as one nurse put it, ‘thinking along their lines'—to understand what was important to them. Appreciating a patient's experience and needs helped the nurses to focus their practice on things that helped to maintain the patient's normal life:

It's the patient and the concerns of the carers that is our number one priority, regardless of the disease process. We say to them, ‘What is your most concerning issue today?’ If these spots on the feet are the first thing that they want to list, then that has to become my first area of concern… regardless of whether I think it's important or not.

The nurses discussed how appreciating a patient's norms was predicated on not taking a patient's circumstance for granted. Instead, they placed equal weight on different forms of expertise, including nursing knowledge, medical knowledge, and lived experience:

a brilliant person asks questions and never assumes… brilliance is about… knowing what to ask, in what context, and not pretending… you're the expert.

**Figure 2:**
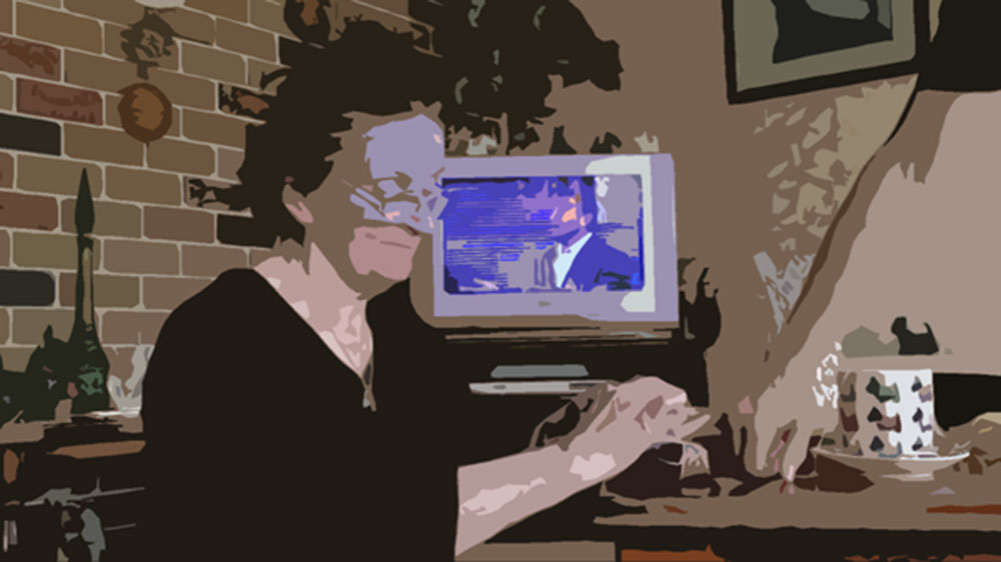
A bereavement visit.

Ultimately, the nurses aimed to be a calming and helpful presence in a patient's home, without causing disruption or concern. A metaphor for this kind of comfortable helpful presence was on display during one reflexive session, which featured a video‐recording of a bereavement visit—a visit to a patient's family following their death—with a patient's widow (see Figure 2). The recording showed the widow wrapping a small gift—a boxed piece of jewellery—while discussing her husband's death and the impact it had on her. One nurse summarised the delicate moment the visiting nurse offered to assist:

When she was tying up the ribbon on the box… she was tying it and untying it; tying it and untying it… [The nurse] said, ‘Do you want me to put my finger there so you can tie it up?’… Just little things like that… come with caring, empathising, and I just thought that was beautiful. That lady… at that moment, all she needed was that presence… We don't have to say much; we just have to listen, be there. And just offering to put her finger there so she could actually tie the ribbon to me was so symbolic of what a bereavement visit should be like.

Time spent with patients and their carers took on more importance for the nurses than technical clinical interventions. Brilliance was found in the calm, quiet, normal moments when patients' and carers' lives were privileged.

## 4 DISCUSSION

Palliative care represents an international priority—this partly follows ageing populations^2^ and the prevalence of chronic health issues,^3^ worldwide. Despite its importance, palliative care is often portrayed negatively in research^12^ and media reports.^17^

To redress this imbalanced focus on all that is wrong with palliative care, and build on scholarship on brilliant palliative care,^23^ this study presents a novel analytical approach—teleological analysis—to clarify what brilliant community‐based palliative care nursing supports and promotes. While previous research highlighted some of the ingredients associated with brilliant palliative care—including ‘anticipatory aptitude and action; a weave of commitment; flexible adaptability; and/or team capacity‐building’—this study established that brilliant community‐based palliative care nursing largely involved maintaining normality in patients’ and carers’ lives. The nurses demonstrated this by masking the clinical aspects of their role, normalising these aspects, and appreciating alternative ‘normals’. This demonstrates an extension of previous findings on brilliant palliative care.

The centrality of normality aligns with some of the literature on palliative care.^48^ For instance, the findings align with Li's^49^ research which argued that niceness can foster a comfortable and ‘socially orderly’,^49^ or normal environment for palliative care patients. Li focussed on the techniques of ‘symbiotic niceness’, which included compliments and humour, to establish friendly and informal atmospheres in interaction. Likewise, Seipp and colleagues’^50^ study of success in specialised palliative homecare found that a sense of security for patients and relatives was key to enabling care at home. This sense of security was fostered by clinician availability, anticipatory clinician actions, and clinician awareness of patient needs and desires. The research focussed on the identification of patient needs and desires by palliative care nurses. In designing and validating a palliative care quality scale, Zulueta and colleagues^51^ noted that to provide good care, it is necessary to identify the needs and desires of the terminal patient, requiring nurses to be self‐aware. While the findings presented in this article align with this observation, they also provoke a more complex requirement, whereby community‐based palliative care nurses know when and how to introduce the clinical aspects of their practice to patients’ and carers’ lived experiences of a life‐limiting illness. Consider the findings in this article, including the art of conversational patient assessment and preparing a Movicol ‘milkshake’ for a patient with constipation.

The findings in this article have implications for those who manage, deliver, and/or research palliative care, particularly that which is community‐based. For managers and clinicians, given the importance of subjugating the clinical aspects of community‐based palliative care, technical clinical interventions should be reconsidered to enable their compatibility with normality in patients’ lives when at home. Take the syringe driver as an example—a bulky item with an overt clinical look. Patients were required to carry it around with them throughout their day (unless bedbound). Given the findings in this article, it might be helpful to redesign home‐based clinical interventions—like the syringe diver—to make them fit better with a patient's life at home. Other simple approaches in day‐to‐day clinical practice might involve moderating clinical conversations with everyday colloquial language, pleasantries, and humour, or ensuring discussion of nonclinical topics to bookend a home visit.

For researchers, given the aforesaid practical implications, there is an opportunity to work with managers, clinicians, patients, and carers to co‐design home‐based clinical interventions and conversational styles of patient assessment and test these to determine whether and how they support and promote brilliant community‐based palliative care nursing. Furthermore, given the novelty of the teleological analysis in this article, there is an opportunity for researchers to use this approach to ask, not only what brilliance in healthcare, more generally, looks like, but also what it ultimately supported and promoted. This would require researchers to question the theoretical endpoint of data about brilliant clinical practices: abductive reasoning asking, where does this brilliant practice take this clinical, organisational, or collegial relationship?

A key strength of this study is approaching community‐based palliative care with a focus on brilliant nursing practices. The nurses in this study noted they often did not have the opportunity to reflect on what they do well. Rather, they typically considered their interactions with patients and families as standard care. Using POSH‐VRE afforded rich data to closely explore the theoretical endpoints of brilliance. Furthermore, based on the joy and satisfaction that emanated from the reflexive sessions, this approach positively impacted the nurses, enabling them to acknowledge and enhance their care of patients and carers. Positive emotions in clinical contexts can have powerful effects, creating new knowledge or altering existing knowledge in a mutually sympathetic upward spiral.^52^ Another strength of this study is it democratised scholarship—rather than position the researchers as the transmitters of knowledge to the co‐researchers as acquirers of this knowledge, the study moved empirical research from the sole clutches of academics and made it accessible to people beyond the academy.^53^ Learning and knowing was thus a process of co‐construction, whereby they collectively engaged in ‘an activity involving increased access to participating roles in expert performance’.^54^ It was an intentional process of ‘listening to the world, of having a concern for the world, of caring for the world, and perhaps even of carrying (the weight of) the world’.^55^

Despite the scholarly contributions offered by this study, two methodological limitations warrant mention. First, because it was not feasible to involve patients and carers in the reflexive sessions with the nurses to examine and understand brilliant palliative care, there are no claims that the research team's fluid understanding of brilliant palliative care nursing reflects that of patients and/or carers. Thus, appreciating the situated nature of brilliance might require different processes with patients and carers, than that described in this article. Second, this study involved one community health centre that offered palliative care—thus, there are no claims the findings can be generalised elsewhere. Therefore, there is an opportunity for future research to consider what brilliant practices supported and promoted, in different contexts, within and beyond Australia or community‐based palliative care.

This study used POSH‐VRE to redress the scholarly preoccupation with gaps, issues, and problems in palliative care, purposely considering brilliant community‐based palliative care nursing. By analysing the data, teleologically, this article demonstrates how the ordinary can be extraordinary. Given the intrusiveness and abnormalising effects of clinical interventions, brilliant community‐based palliative care can be realised when nurses enact practices that serve to return a patient or carer to normality.

## AUTHOR CONTRIBUTIONS

Ann Dadich and Aileen Collier conceived and designed the study. Ann Dadich, Michael Hodgins, and Aileen Collier lead the developed of the article; and all authors contributed to the collection, analysis, and interpretation of the data, as well as the finalisation of the article.

## CONFLICT OF INTEREST STATEMENT

The authors declare no conflict of interest.

## ETHICS STATEMENT

This study was approved by the South Western Sydney Local Health District Human Research Ethics Committee (reference number: HREC/15/LPOOL/73). All participants indicated informed consent.

## Data Availability

Data are unavailable due to ethical restrictions.
